# Comparing Tobacco and Alcohol Policies From a Health Systems Perspective: The Cases of the Philippines and Singapore

**DOI:** 10.3389/ijph.2022.1605050

**Published:** 2022-10-13

**Authors:** Gianna Gayle Herrera Amul, Jean-Francois Etter

**Affiliations:** ^1^ Institute of Global Health, Faculty of Medicine, Université de Genève, Geneva, Switzerland; ^2^ Research for Impact Singapore, Singapore, Singapore; ^3^ School of Government, Ateneo de Manila University, Quezon City, Philippines

**Keywords:** tobacco control, health systems, alcohol control, Philippines, Singapore, health policy, public health law, policy surveillance

## Abstract

**Objective:** To provide a comparative analysis of current tobacco and alcohol control laws and policies in the Philippines and Singapore

**Methods:** We used a public health law framework that incorporates a systems approach using a scorecard to assess the progress of the Philippines and Singapore in tobacco and alcohol control according to SDG indicators, the WHO Framework Convention on Tobacco Control and the WHO Global Strategy to Reduce Harmful Use of Alcohol. We collected data from the scientific literature and government documents.

**Results:** Despite health system differences, both the Philippines (73.5) and Singapore (86.5) scored high for tobacco control, but both countries received weak and moderate scores for alcohol control: the Philippines (34) and Singapore (52.5). Both countries have policy avenues to reinforce restrictions on marketing and corporate social responsibility programs, protect policies from the influence of the industry, and reinforce tobacco cessation and preventive measures against alcohol harms.

**Conclusion:** Using a health system-based scorecard for policy surveillance in alcohol and tobacco control helped set policy benchmarks, showed the gaps and opportunities in these two countries, and identified avenues for strengthening current policies.

## Introduction

The burden of noncommunicable diseases (NCDs) is now pervasive in both high-income and low- and middle-income economies. In a high-income country like Singapore, NCDs account for an estimated 84% of the burden of disease and about 83% of deaths, and even in a lower-middle-income country like the Philippines, NCDs already account for more than 64% of the burden of disease and 70% of deaths [1]. According to the Global Burden of Disease study, tobacco and alcohol use remained the top risk factors for disease and death burden in the Philippines and Singapore since 1990, for both males and females [2]. When disaggregated by gender, males bear a higher tobacco-attributable and alcohol-attributable burden of disease than females in both the Philippines and Singapore [2].

From a global health perspective, the World Health Organization (WHO) Framework Convention on Tobacco Control (FCTC) has been a powerful legal framework and a foundation for the development and implementation of tobacco control policies in countries at various economic development levels [3, 4]. While tobacco control implementation has been assessed using the World Bank’s Tobacco Control Scale, the WHO FCTC and health systems frameworks, alcohol control policies vis-à-vis the WHO Global Strategy to Reduce Harmful Use of Alcohol (Global Alcohol Strategy hereafter) have yet to be assessed using a systems perspective [5–9].

The methods of public health law, including policy surveillance, have also been used to assess tobacco and alcohol measures, and have been invariably adopted by the WHO in monitoring international health law, including the FCTC [10, 11]. Despite the growth in comparative mechanisms, alcohol policy surveillance has yet to be institutionally adapted for the rigorous evaluation of legislation for alcohol control in any of the member states of the Association of Southeast Asian Nations (ASEAN).

The Philippines and Singapore offer case studies of two different health systems in ASEAN with recent reforms in their tobacco and alcohol control policies. While both countries face the increasing burden of NCDs and the expansion of the alcohol and tobacco industry [12], both countries offer policy lessons on alcohol and tobacco control towards the development of a regional framework for alcohol control [13].

This study aims to provide a comparative analysis of tobacco and alcohol policies in the Philippines and Singapore from a public health law and systems approach. This study offers a comparative policy surveillance framework that acknowledges the complexity of both alcohol and tobacco control and assesses countries on their progress in tobacco and alcohol control beyond demand and supply-reduction measures, by looking into the WHO health system building blocks—with particular focus on leadership and governance, financing, human resources, information, service delivery and access to essential medicines [8].

## Methods

We used a systems approach to develop a scorecard measuring the progress of the Philippines and Singapore in tobacco and alcohol control, based on the WHO’s health system’s six building blocks (leadership and governance, financing, human resources, information, service delivery and medical products and technologies) vis-à-vis Sustainable Development Goal 3 (Health and Well-Being) outcome indicators, the FCTC, and the Global Alcohol Strategy [14–17].

We drew on methodology from transdisciplinary public health law, particularly policy surveillance, which involves the empirical tracking of law and policies of disease, and global health law which focuses on international law and health [11, 18].

For the policy surveillance, we conducted an online document search to include official English versions of policy documents (legislation, implementing rules and subsidiary regulations and related guidelines specific to tobacco and alcohol) and reports from the websites of the WHO, websites of various Philippine and Singapore government agencies and online policy databases. Both the Philippines and Singapore use English as one of their official languages. These were cross-checked with official English versions in the Singapore Statutes Online and in the Philippines’ Official Gazette available online. We supplemented this with reports in English from non-governmental organisations, corporate documents and news articles.

Additionally, we supplemented this with a review of the peer-reviewed literature in English on PubMed published from January 2009 to December 2020. Please see Supplementary Figure S1 for the search strategy. We included articles that specifically refer to alcohol and tobacco policies in the Philippines and Singapore. We excluded epidemiological, clinical, and behavioural studies with no reference to alcohol or tobacco policies in the Philippines and Singapore.

### Scorecard

We adapted the tobacco control scorecard and the indicators developed by Amul and Pang [8]. They assessed the implementation of tobacco control using the health system building blocks by assigning scores for each article in the WHO FCTC, with the highest scores (10 points) allotted for MPOWER measures which include monitoring tobacco use, protecting people from tobacco smoke, offering help to quit tobacco, warning about the dangers of tobacco, enforcing tobacco advertising, promotion and sponsorship (TAPS) bans, and raising taxes on tobacco (See Table 1). The scorecard incorporated indicators from the FCTC Implementation Database and the WHO Global Health Observatory.

**TABLE 1:** Scoring framework for the tobacco control scorecard based on the World Health Organization Framework Convention on Tobacco Control according to the health system building blocks (Singapore and the Philippines, 2022)^a^.

Health system building block	Framework convention on tobacco control article	Indicator	Score
Leadership and governance (65)	Article 5.1. Development, implementation, updating and review of multisectoral national tobacco control strategies	Multisectoral national tobacco control strategy	2.5
	Article 5.2. Establishing, reinforcing, financing a national coordinating mechanism or focal points for tobacco control	National coordinating mechanism or focal point for tobacco control	2.5
	Article 5.3. Protecting public health policies from the commercial and vested interests of the tobacco industry	Whole-of-government code of conduct/non-interference policy	5
	Article 6. Price and tax measures to reduce demand for tobacco	At least 75% excise tax share on final price	10
	Article 8. Protection from tobacco smoke	Compliance with regulations on smoke-free environments	10
	Article 11. Packaging and labelling of tobacco products	At least 50% of package consists of large graphic health warnings	10
	Article 12. Education, communication, training and public awareness	Anti-tobacco mass media campaigns	5
	Article 13. Tobacco advertising, promotion and sponsorship	Complete ban on direct tobacco advertising	5
		Complete ban on tobacco promotion and sponsorship	5
	Article 15. Illicit trade in tobacco products	Tracking regime to further secure the distribution system	5
	Article 16. Sales to and by minors	Sales to minors prohibited	2.5
	Article 17. Tobacco growing and support for economically viable alternatives	Viable alternatives provided to tobacco growers	2.5
Financing (10)	Article 26. Financial resources	At least USD 0.11 government expenditure on tobacco control per capita	5
		National health insurance covers cost of smoking cessation support	2.5
		National health insurance covers cost of NRT	2.5
Service delivery (10)	Article 14. Demand-reduction measures concerning tobacco dependence and cessation	Toll-free quitline/helpline	5
		Availability of smoking cessation support in any facility (primary care, hospitals, health clinics, community)	5
Information (5)	Article 20. Research, surveillance and exchange of information	Recent, representative and periodic (at intervals of five years or less) data for both adults and youth	3
	Article 21. Reporting and exchange of information	Periodic reports to the FCTC Secretariat (every two years)	2
Human resources (5)	Article 12d. Training or sensitization and awareness programmes on tobacco control for health workers, community workers, social workers, media professionals, educators, decision makers, administrators and other concerned persons	Full-time staff for tobacco control	2
		Training on tobacco control for health workers, community workers, social workers, media professionals, educators, decision makers, administrators and others	3
Medical products, vaccines and technologies (5)	Article 14.2d. Facilitating accessibility and affordability of pharmaceutical products for the treatment of tobacco dependence	Nicotine replacement therapy is in the country’s essential drug list or publicly available	2
		Nicotine replacement therapy free or reimbursable	3
		Maximum score	100

a This scoring framework is adapted from Amul and Pang [8] which is a modified version of the European Tobacco Control Scale and the Southeast Asia Tobacco Control Alliance Framework Convention Tobacco Control Scorecard and included indicators from the World Health Organization Report on the Global Tobacco Epidemic, the World Health Organization Global Health Observatory [8].

Existing alcohol policy assessment tools focused only on five domains—physical availability, drinking context, alcohol prices, alcohol advertising and drivers of motor vehicles [19–21]. A detailed AAPS policy scorecard for Southeast Asia is also incorporated into the scorecard for alcohol control policies [12]. For the alcohol control scorecard, we incorporated indicators and also compiled policy data (where available) from the WHO Global Information System for Alcohol and Health, and the country profiles in the most recent WHO Global Status Report on Alcohol and Health [22, 23]. We assigned scores for each policy recommendation in the Global Alcohol Strategy, with the highest scores (10 points) allotted for measures in the WHO SAFER Initiative (SAFER) [24]. These policies included restrictions on alcohol availability, drink-driving countermeasures, access to screening, brief interventions and treatment, restrictions on alcohol advertising, promotion and sponsorship (AAPS), and raising alcohol prices through excise taxes and other pricing policies [24] (See Table 2).

**TABLE 2:** Scoring framework for the alcohol control scorecard based on the World Health Organization Global Strategy to Reduce Harmful Use of Alcohol according to the health systems building blocks (Singapore and the Philippines, 2022)^a^.

Health systems building block	World Health Organization Global Strategy to Reduce the Harmful Use of Alcohol	Indicators	Score
Leadership and Governance (73)	Alcohol control measures must be guided and formulated by public health interests and protected from industry interference and commercial interests	Whole-of-government written code of conduct or non-interference policy (*proxy indicator*)	5
	Area 1. Leadership, awareness, and commitment (15)	National, subnational strategies, plans of action and activities	5
		• Written national policy	
		• National action plan	
		Establishment of implementing institution or agency	0.5
		Coordination with other relevant sectors	5
		Access to information, effective education, and public awareness of alcohol-related harms	2
		Raising awareness of harm to others	2.5
		• Presence of awareness-raising activities	
	Area 3. Community Action	Community mobilization to prevent under-age drinking and develop alcohol-free environments	1
		• National support for community action	
	Area 4. Drink-driving policies and countermeasures	National minimum legal blood alcohol concentration when driving a vehicle	1
		sobriety checkpoints and random breath testing	1
		administrative suspension of driving licences	1
		graduated licensing for novice drivers	1
		ignition interlocks	1
		mandatory driver education, counselling, and treatment	2
		availability of alternative transportation in drinking places	1
		public awareness and information campaigns	1
		targeted mass media campaigns (youth events, holidays)	1
	Area 5. Availability of alcohol	Legislation to prevent illegal alcohol production	3
		• National control of production, import, sale, distribution and export (through government monopoly or through licensing)	
		Legislation to prevent illegal alcohol sale	3
		Appropriate minimum age for purchase and consumption of alcohol	2
		• National legal minimum age for on-/off-premise sales of alcoholic beverages	
		Prevent sales to intoxicated persons and those below legal age	2
		• Restrictions for on−/−off premise sales of alcoholic beverages	
	Area 6. Marketing of alcoholic beverages (10)^a^	Regulatory frameworks based on legislation for alcohol marketing	6
		• Legally binding regulations on alcohol advertising (beer, wine, spirits)	
		• Legally binding regulations on product placement (beer, wine, spirits)	
		• Legally binding regulations on alcohol sponsorship (beer, wine, spirits)	
		• Legally binding regulations on sales promotion (beer, wine, spirits)	
		Development of public agencies for systems of surveillance of alcohol marketing	2
		Administrative and deterrence systems for infringement on marketing restrictions	2
	Area 7. Pricing policies (10)	Domestic taxation	3
		• Excise tax on beer, wine, spirits	
		Regular price review	3
		• Inflation adjustment on alcohol taxes	
		Price measures other than taxation	4
		• Banning of price promotions, discounts, sales below costs, flat rates for unlimited drinking and other volume sales (1)	
		• Minimum alcohol pricing (1)	
		• Price incentives for non-alcoholic beverages (1)	
		• Reducing subsidies to economic operators in alcohol (1)	
	Area 8. Reducing the negative consequences of drinking and alcohol intoxication) (7)	Regulating drinking context to minimize violence	1
		Laws against serving to intoxication and legal liabilities	1
		Management policies on server training	0.5
		• Systematic alcohol server training	
		Reducing alcoholic strength	0.5
		Care or shelter for severely intoxicated people	0.5
		Providing consumer information and labelling alcoholic beverages on alcohol-related harms	3.5
		• Legally required health warning labels on alcohol advertisements and/or on alcohol containers	
		• Requirement to display consumer information about calories, additives, vitamins and micro-elements on the labels of alcohol containers	
		• Number of standard alcoholic drinks displayed on containers	
		• Alcohol content displayed on containers	
	Area 9. Reducing the public health impact of illicit alcohol and informally produced alcohol (5)	Licensing regimes on production and distribution of alcoholic beverages	2.5
		• Legislation to prevent the illegal production of alcohol (beer, wine, spirits)	
		• Legislation to prevent the illegal sale of alcohol (beer, wine, spirits)	
		Regulation on sales of informally produced alcohol	0.5
		Control and enforcement system (tax stamps)	0.5
		Tracking and tracing systems for illicit alcohol	0.5
		Cooperation in combating illicit alcohol	0.5
		Public warnings about contaminants and health threats from informal or illicit alcohol	0.5
Health Service Delivery (10)	Area 2. Health services’ response (9)	Increasing capacity for health and social welfare systems for prevention, treatment and care for alcohol use disorders	2
		Supporting initiatives for screening and brief interventions for hazardous and harmful drinking at primary health care settings & early identification and management of harmful drinking among pregnant women	2
		Improving capacity for prevention, identification and interventions for families and individuals living with foetal alcohol syndrome	1
		Coordination of integrated prevention, treatment and care strategies and services for alcohol use disorders and comorbid conditions	1
		System of registration and monitoring of alcohol-attributable mortality and morbidity with regular reporting	1
		Culturally sensitive health and social services	1
		Securing and enhancing availability, accessibility, and affordability of treatment services for groups of low socioeconomic status	1
	Area 3. Community Action	Providing community care and support for affected individuals and their families	1
Information (5)	Area 10. Monitoring and Surveillance (5)	Framework and systems for monitoring alcohol consumption, and alcohol-related harm	2
		• National monitoring system for alcohol consumption	
		• National monitoring system for health consequences of alcohol	
		• National monitoring system for social consequences of alcohol	
		• National monitoring system for alcohol policy responses	
		National entity for monitoring alcohol	0.5
		Common set of indicators for tracking harmful use of alcohol and policy responses	0.5
		• National surveys where alcohol is specifically addressed or part of a larger international survey	
		Data repository based on internationally agreed indicators	1
		Policy evaluation mechanisms	1
Human Resources (5)	Area 3. Community Action (5)	Rapid assessment of gaps and priority areas for intervention	0.5
		Facilitating recognition of alcohol-related harms at the local level and promoting responses to local determinants	1
		Strengthening the capacity of local authorities	1
		Providing information on effective community-based interventions and building capacities at the community level	1
		Developing community programs	1.5
Financing (4)	Mobilizing resources/funding for prevention, treatment, and rehabilitation (*proxy indicators*)	Mobilizing resources/funding for prevention	2
		Mobilizing resources/funding for treatment	1
		Mobilizing resources/funding for rehabilitation	1
Access to Essential Medicines (3)	Availability of essential medicines for alcohol use disorders and alcohol dependence (*proxy indicators*)	Availability of naltrexone in the national Essential Medicines List	1
		Availability of acamprosate in the national Essential Medicines List	1
		Availability of disulfiram in the national Essential Medicines List	1
		Maximum score	100

a This scoring framework used relevant indicators from the World Health Organization Global Information System for Alcohol and Health which is a component of the World Health Organization Global Health Observatory, and the World Health Organization Global Status Report on Alcohol and Health.

To incorporate policies that go beyond the health system for implementation and enforcement including taxation, illicit trade, marketing restrictions, community action, smoke-free environments, and drunk-driving countermeasures, we adapted the concept of health system governance as a process that involves “ensuring strategic policy frameworks exist and are combined with effective oversight, coalition-building, regulation, attention to system design and accountability” and is determined by the interaction of the State, health service providers, and citizens [25]. Additionally, we also adapted the principle of health in all policies which recognize “the policy practice of including, integrating or internalizing health in other policies that shape or influence the social determinants of health [26]”.

GGA devised the scoring system based on an existing tobacco control scorecard which used the WHO health system building blocks as a framework [8]. GGA compiled the policy data from the document search and allotted the scores for each country. Based on the results of the document search for policy data, GGA generated the scores for each policy in each country based on the extracted policy data. Each indicator has allotted points and when there are policy data that meets the indicator’s full scope, a full score is tabulated for that indicator. When the policy only covers a partial scope of the indicator, the tally of points scored for each partial scope is tabulated. When there is no policy for that indicator, no points are tabulated for that indicator. Total scores for both the tobacco and alcohol control scorecard range from 0 to 100. An overall score between 1 and 25 is categorized as poor, between 26 and 50 is weak, between 51 and 75 is moderate, and between 76 and 100 is strong.

Tables 1, 2 show the breakdown of the indicators and the scoring system used in this study. There were fewer data sources for alcohol control than for tobacco control, and where data is not available, we used proxy indicators for financing and access to essential medicines.

## Results

In the peer-reviewed literature, we found 93 articles on tobacco control and 94 articles on alcohol control in the Philippines, and 200 articles on tobacco control and 139 articles on alcohol control in Singapore. We used Endnote to compile all search results for various combinations of the search terms and removed duplicates using the ‘find duplicates’ function; after screening the remaining 211 titles and abstracts for articles using the specified inclusion and exclusion criteria, we retained 24 articles on Singapore and 17 articles on the Philippines for inclusion in the qualitative synthesis.

Combining results from the literature and policy data from the document search, the next section offers a snapshot of the policy framework for alcohol and tobacco control in each country, followed by a narrative synthesis based on each country’s strengths and gaps in the health system building blocks, and a discussion on avenues of intervention for both countries.

### Policy Framework

The Philippines and Singapore are both parties to the FCTC and both have selectively implemented some policy recommendations from the Global Alcohol Strategy [15, 16, 27]. However, both countries have yet to ratify the FCTC Protocol to Eliminate Illicit Trade in Tobacco Products (hereafter “Protocol on illicit trade’’), which came into force in 2018, and they have not yet announced any plans to do so [28]. Table 3 and the following sections show that both countries have implemented most of the WHO FCTC measures [29–32]. Table 4 shows that both countries have implemented only a selection of recommendations from the Global Alcohol Strategy, with a particular focus on alcohol taxation and drunk-driving prevention measures [22]. Both countries implement surveillance on tobacco use; both participate in the Global Tobacco Surveillance System [33]. Both countries also report to the WHO for the Global Status Report on Alcohol and Health, albeit these reports show limited surveillance in both prevalence and policy [22]. Tables 3, 4 also show that the Philippines has strong tobacco control, but weak alcohol control. Singapore scored strongly on all health system building blocks for tobacco control but obtained moderate scores for alcohol control.

**TABLE 3:** Tobacco control score card for the Philippines and Singapore, based on the World Health Organization Framework Convention on Tobacco Control with the health systems building blocks as a framework (Philippines and Singapore, 2020).

Health systems building blocks	World Health Organization Framework Convention on Tobacco Control (corresponding points)	Philippines^a^	Singapore^b^
Leadership and Governance	Leadership and governance sub-total	**53**	**55.5**
	Article 5.1. Development, implementation, updating and review of multisectoral national tobacco control strategies (2.5)	2.5	2.5
	Article 5.2. Establishing, reinforcing, financing a national coordinating mechanism or focal points for tobacco control (2.5)	2.5	2.5
	Article 5.3. Protecting public health policies from the commercial and vested interests of the tobacco industry (5)	5	5
	Article 6. Price and tax measures to reduce demand for tobacco (10)	7	8
	Article 8. Protection from tobacco smoke (10)	10	5
	Article 11. Packaging and labelling of tobacco products (10)	10	10
	Article 12. Education, communication, training, and public awareness (5)	5	5
	Article 13. Tobacco advertising, promotion, and sponsorship (10)	6	10
	Article 15. Illicit trade in tobacco products (5)	5	0
	Article 16. Sales to and by minors (2.5)	2.5	2.5
	Article 17. Tobacco growing and support for economically viable alternatives (2.5)	2.5	NA
Financing	Article 26. Financial resources (10)	**3.5**	**9**
Health Service Delivery	Article 14. Demand-reduction measures concerning tobacco dependence and cessation (10)	**7**	**8**
Information	Information sub-total	**5**	**5**
	Article 20. Research, surveillance, and exchange of information (3)	3	3
	Article 21. Reporting and exchange of information (2)	2	2
Human Resources	Article 12(d). Training or sensitization and awareness programs on tobacco control for health workers, social workers, media professionals, educators, decision-makers, administrators, and other concerned persons (5)	**3.5**	**5**
Access to Essential Medicines	Article 14.2d. Facilitating accessibility and affordability of pharmaceutical products for the treatment of tobacco dependence (5)	**1.5**	**4**
Total Score^c^ (100)		**73.5**	**86.5**

a Scores are based on policy data from the World Health Organization Framework Convention on Tobacco Control Philippines Report 2018 and 2020 and cross-checked with reported legislation [8, 32, 33].

b Scores are based on policy data from the World Health Organization Framework Convention on Tobacco Control Singapore Report 2018 and 2020 and cross-checked with reported legislation [8, 30, 31].

c An overall score between 1 and 25 is categorized as poor, between 26 and 50 is weak, between 51 and 75 is moderate, and between 76 and 100 is strong.

Specific subtotals and the total values are highlighted in bold.

**TABLE 4:** Alcohol control score card for the Philippines and Singapore, based on the World Health Organization Global strategy to reduce the harmful use of alcohol with the health systems building blocks as a framework (Philippines and Singapore, 2020).

Health systems building blocks	WHO Global Strategy to Reduce the Harmful Use of Alcohol (corresponding points)	Philippines^a^	Singapore^a^
Leadership and governance	Leadership and governance sub-total	**26**	**34.5**
	Alcohol control measures must be guided and formulated by public health interests and protected from industry interference and commercial interests (5)	0	0
	Area 1. Leadership, awareness, and commitment (15)	5	8.5
	Area 3. Community Action (1)	0.5	0.5
	• Community mobilization to prevent under-age drinking and develop alcohol-free environments		
	Area 4. Drink-driving policies and countermeasures (10)	4	4.5
	Area 5. Availability of alcohol (10)	8	10
	Area 6. Marketing of alcoholic beverages (10) ^b^	0.5	1
	Area 7. Pricing policies (10)	3	4
	Area 8. Reducing the negative consequences of drinking and alcohol intoxication) (7)	1	1.5
	Area 9. Reducing the public health impact of illicit alcohol and informally produced alcohol (5)	4	4.5
Health Service Delivery	Health service delivery sub-total	**3**	**6**
	Area 2. Health services’ response (9)	3	5
	Area 3. Community Action	0	1
	• Providing community care and support for affected individuals and their families (1)		
Information	Area 10. Monitoring and Surveillance (5)	**2**	**2.5**
Human Resources	Area 3. Community Action (5)	**1**	**3.5**
	• Rapid assessment of gaps and priority areas for intervention (0.5); facilitating recognition of alcohol-related harms at the local level and promoting responses to local determinants (1); strengthening the capacity of local authorities (1); providing information on effective community-based interventions and building capacities at the community level (1); developing community programs (1.5)		
Financing	*Proxy indicator:* Mobilizing resources/funding for prevention (2), treatment (1) and rehabilitation (1)	**1**	**3**
Access to Essential Medicines	*Proxy indicator:* Availability of essential medicines for alcohol use disorders and alcohol dependence (3)	**1**	**3**
Total Score (100)^c^		**34**	**52.5**

a Scores are based on policy data from the World Health Organization Global Information System on Alcohol and Health 2016 and cross-checked with relevant legislation [63].

b Index score based on Alcohol Advertising, Promotion and Sponsorship (AAPS) Policy Scorecard by Amul [12].

c An overall score between 1 and 25 is categorized as poor, between 26 and 50 is weak, between 51 and 75 is moderate, and between 76 and 100 is strong.

Specific subtotals and the total values are highlighted in bold.

**FIGURE 1:**
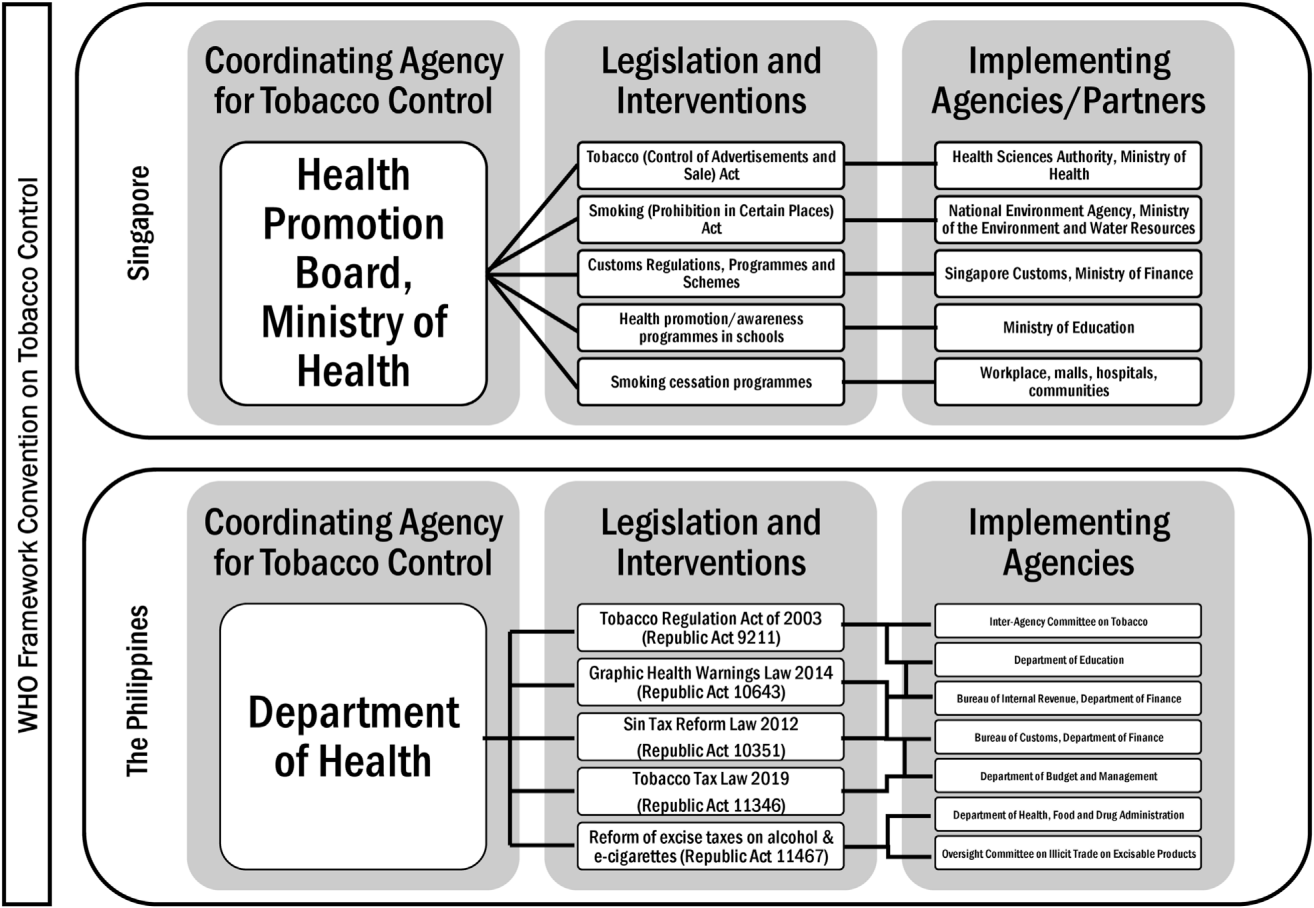
Tobacco control in Singapore and the Philippines (Singapore and the Philippines, 2020).

**FIGURE 2:**
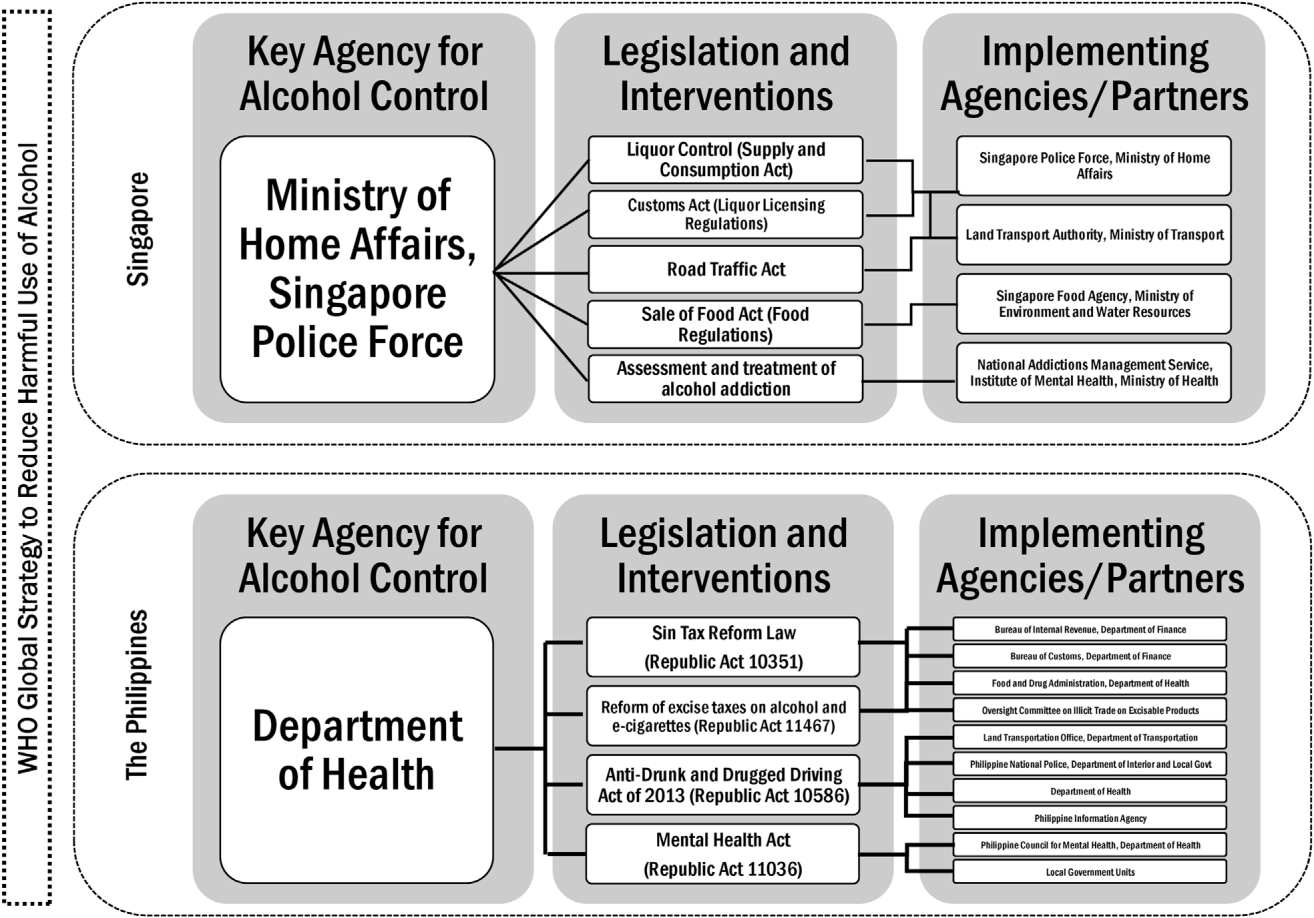
Alcohol control in Singapore and the Philippines (Singapore and the Philippines, 2020).

Singapore is historically a strong authoritarian state, and this translates to strong political will and leadership in terms of tobacco control, which began in the 1970s, while alcohol control has mainly been focused on price measures, including taxes and tariffs (Supplementary Table S1), and recently on reducing accessibility, with licensing (Supplementary Table S3), no-liquor zones and sale restrictions, and increasing penalties for drunk driving [34–36]. Figure 1 presents the laws and implementing agencies that govern tobacco control policies in the Philippines and Singapore [37]. Only in the past decade did the Philippine government (particularly the Presidency) show leadership in terms of both tobacco and alcohol control, with consecutive reforms on alcohol taxation and anti-drunk driving laws (Supplementary Tables S2, S4) [38]. Figure 2 shows the legislation and responsible agencies that implement the policies for alcohol control in the Philippines and Singapore [39].

### Strengths in Tobacco Control

Table 3 shows that both countries score relatively high on the tobacco control scorecard, but that Singapore (86.5) scored higher than the Philippines (76.5). Singapore’s strengths lie in the strict enforcement of its tobacco control policies including tobacco taxation policies, financing of health promotion, smoke-free policies, a comprehensive ban on tobacco advertising, promotion, and sponsorship (TAPS hereafter), packaging and labelling measures (standardised packaging), and access to essential medicines and therapies for tobacco cessation [37, 40] (Table 3). Singapore adjusts its tax rates according to inflation, and it increased its tobacco tax rate to 67.5% in 2018, while it increased alcohol tax rates to SGD88 per litre in 2014 [41]. Singapore has comprehensively banned advertising, promotion, and sponsorship of tobacco products (including e-cigarettes) [42]. Singapore has also progressively raised the minimum legal age for smoking to 21 years [43] and banned the import, distribution, sale or offer for sale of cigarette packs that contain less than 20 sticks, and it does not have any duty-free concessions or goods and services tax relief for cigarettes [37, 44].

Both countries have implemented several measures in compliance with the FCTC article on illicit trade, including the Singapore Duty-Paid Cigarette (SDPC) markings and the Philippines’ tax stamps under the Internal Revenue Stamps Integrated System (IRSIS) to be affixed on all unit packets of cigarettes and alcohol products in 2018 [8, 37].

Singapore also scores high in terms of access to essential medicines, and nicotine replacement therapy, bupropion and varenicline (medications to treat tobacco dependence) are part of Singapore’s essential medicines list (EML), while only varenicline is part of the Philippines’ EML [45, 46]. Nicotine replacement therapy is free or reimbursable through the public health sector in Singapore but not in the Philippines [8, 40]. Singapore’s Health Promotion Board implements public education campaigns that complement multi-sectoral and community-based national smoking cessation programmes [47].

The Philippines’ strengths in tobacco control lie in its tobacco taxation policies (Supplementary Table S1) and its explicit policy of protecting the public administration from tobacco industry interference [48, 49] (Table 3). The Philippines’ recent tax reforms have set tax rates that increase every year from 2020 to 2024, after which tax rates are set to increase annually by 5% for tobacco products and 6% for alcohol products [38].

### Gaps in Tobacco Control

Singapore’s weakness in tobacco control lies in the lack of explicit, publicly available guidelines to protect public policies from industry interference, despite the city-state’s otherwise strong anti-corruption measures. Singapore implements a “government-wide code of conduct and internal guidelines for relevant agencies’ governing interaction with the tobacco industry,” but there is no publicly available written policy about these guidelines [30]. Additionally, Singapore’s *Prevention of Corruption Act* covers such interactions in both the public and private sectors [50].

The Philippines’ key weakness in tobacco control lies in the inclusion of the tobacco industry in the Philippines’ Inter-Agency Committee on Tobacco because this creates a conflict of interest. The Philippines became a party to the FCTC only after legislation established this tobacco control policy-making committee that includes the tobacco industry, which is an infringement of Article 5.3 of the FCTC that obligates the Philippines as a party to the FCTC to protect tobacco control policies from commercial interests of the tobacco industry [51].

Additionally, both countries have weaknesses in terms of illicit tobacco trade control, and both have yet to ratify the FCTC Protocol on illicit trade. The Philippines has less comprehensive tobacco marketing restrictions, and there are still loopholes for the protection against advertising and promotion, especially at the point of sale, which the tobacco industry exploits [52]. Moreover, the Philippines still tolerates sales of single-stick cigarettes, although the law requires that cigarettes be sold in 20-cigarette packs [53]. Furthermore, the Philippines still allows duty-free concessions on tobacco products [8].

The two countries vary in their approach to electronic nicotine delivery devices (ENDS), with Singapore being comprehensively restrictive—banning emerging and alternative nicotine products, while the Philippines preferred regulation through taxation [32].

### Strengths in Alcohol Control

Singapore (52.5) scored higher than the Philippines (34) on the alcohol control scorecard (Table 4). As shown in Table 4, despite a marked difference in financing capacities, the strengths of Singapore’s alcohol control measures lie in an array of tax measures, licensing regime, restrictions in availability (minimum legal age, zoning, and time of sale), access to essential medicines, and drunk driving prevention measures. Singapore scores high on access to essential medicines for alcohol use disorders. The most common medications for alcohol use disorders and alcohol dependence—naltrexone, acamprosate and disulfiram are available on prescription in Singapore, but not subsidised [54, 55]. Moreover, Singapore’s National Addictions Management Service offers a helpline for those seeking help with their alcohol addiction and runs an inpatient facility and treatment services for adolescents with substance abuse issues [56, 57].

As with tobacco control, the strength of the Philippines’ alcohol control measures particularly lies in its alcohol taxation policy [48]. For tracking and tracing the products (a measure against illicit trade), the Philippines also requires import permits and tax stamps on imported alcoholic beverages. In 2019, to protect children, the government issued guidelines on the commercial display at point-of-sale, and on the sale, promotion, and advertising of alcoholic beverages [58]. In terms of licensing, both Singapore and the Philippines have retail licensing regimes for alcohol, but only Singapore has retail licensing regimes for both tobacco and alcohol.

### Gaps in Alcohol Control

Despite the socio-economic differences between them, both countries share weaknesses in alcohol policies. First, both lack comprehensive and legally binding regulations on alcohol advertising, promotion, and sponsorship; both Singapore and the Philippines have voluntary industry measures that are known to be ineffective and thus both countries have poor scores in policies to regulate alcohol marketing [12] (Table 4).

Second, Singapore and the Philippines also score low on alcohol pricing policies with the lack of minimum pricing, lower pricing of non-alcoholic beverages, below-cost and volume discounts ban, or added levy on specific products. Both countries still have duty-free concessions on alcohol at 2 L per person per trip.

Third, both do not have specific and written guidelines on interaction with the alcohol industry to protect policies from commercial interests, a key element of SAFER [24]. Moreover, despite the conflict of interest, both countries’ governments still engage in public-private partnerships (PPPs) with the alcohol industry and promote the alcohol industry’s corporate social responsibility (CSR) programmes [12].

Fourth, Table 4 also shows that both countries still lack measures that help inform the public about alcohol harms on alcohol product packaging and labelling. The Philippines even lacks harmonization of its alcohol labelling regulations to apply for both local and imported alcoholic beverages [12].

Fifth, the Philippines’ lower scores on alcohol control can be attributed in part to the low access to essential medicines for the treatment of alcohol use disorders, as only naltrexone is listed in its EML [46].

Sixth, both countries have similarly low scores on information because of the lack of a national system for monitoring and surveillance of alcohol harms, despite having national surveys on youth and adult alcohol consumption. Singapore has a national system of epidemiological data collection for alcohol use and health service delivery, but the Philippines does not have any of the two; both do not report data from health services on alcohol use and alcohol use disorders.

Seventh, the Philippines scores low (3) in health service response to harmful alcohol use because of the slow implementation of policies which mandate prevention, treatment, and rehabilitation for alcohol use disorders at the community level [53]. There is room to enhance health service delivery for alcohol use disorders, especially with the predominant public-private referral system for treatment and rehabilitation services for alcohol addiction. In a country of about 100 million, there are only 13 private rehabilitation facilities and one government-run rehabilitation centre for alcohol dependence and alcohol addiction [59].

Finally, as a high-income economy with a developed health system, Singapore does not earmark taxes on tobacco and alcohol for prevention and control measures of these products. However, it has invested in health promotion with an average annual budget of SGD186 million (USD133 million) from 2009 to 2019 [37, 60]. On the other hand, as a lower-middle-income economy, the Philippines, with the 2019 tax reforms (Supplementary Table S4), has earmarked revenue from taxes on alcohol, tobacco, heated tobacco and vapour products, and sweetened alcoholic beverages to fulfil its universal health coverage goals (60%), health infrastructure development (20%), and the SDGs (20%) [48].

Earmarking tax revenue for healthcare from 2004 led to a substantive increase in the Department of Health’s budget, explained by an 87.5% increase in excise tax revenue from alcohol and tobacco from 2015 to 2019 [48, 61]. However, the Philippines still has a low score in financing tobacco and alcohol control because the taxes are not earmarked for this purpose.

## Discussion

In this study, we described the strengths and weaknesses of tobacco and alcohol control policies in Singapore and the Philippines, using the WHO’s health system building blocks as a framework for analysis. Singapore has always considered tobacco control a critical concern for public health, but alcohol control remains primarily an issue of public order and road safety rather than a public health issue. As shown in the policy framework for alcohol control in Singapore in Figure 2, the key agency for alcohol control is the Singapore Police Force, not the Ministry of Health. In the Philippines, while both alcohol and tobacco control are on the public health agenda, alcohol control policies are reliant on alcohol taxes aimed at revenue generation for universal health coverage. The scorecard shows that when assessed by health system building blocks, most of the alcohol control policies in the Philippines are weak, except when recent tax reforms led to an increase in alcohol taxes earmarked for healthcare.

Various tobacco control scorecards have been used to track the implementation of the FCTC, but assessments of alcohol control policies are less comprehensive [9, 62, 63]. This study’s originality lies in its use of health systems as a framework to assess alcohol control policies in two diverse countries [64].

### Avenues for Intervention

The results of the health system scorecard analysis for alcohol and tobacco control suggest various avenues for intervention. First, leadership and governance are critical in tobacco and alcohol control, as the effective implementation of the FCTC and the Global Alcohol Strategy relies on concrete, legally binding and enforceable policy measures [65]. This calls for stronger engagement of various actors—intergovernmental organizations, global health networks, non-government organizations, community organizations, and the academe—to work with governments to pursue, promote and support the implementation of stronger alcohol and tobacco control policies.

Second, given the pervasiveness of self-regulation for the alcohol industry in the Philippines and Singapore and the lack of marketing restrictions, the political influence of the alcohol industry merits a better response from policymakers. This is possible and has been done in Europe and the Americas [12, 66]. This calls for policy approaches that capture the commercial determinants of health [67].

Third, a look into the global policy environment is necessary. While the FCTC requires parties to allot funding for tobacco control, there is no similar financing recommendation for the implementation of the WHO Global Alcohol Strategy. This creates a funding gap for implementing alcohol control policies, in both the Philippines and Singapore.

The two countries diverge in their approach to ENDS with prohibition in Singapore and regulation in the Philippines. While there is initially strong regulation on the minimum legal age of use of ENDS in the Philippines at 21, recent legislation lowered this age to 18, the same minimum legal age for cigarette use in the country. This stands in contrast to the minimum legal age in Singapore where the minimum legal age for the purchase, use, possession, sale, and supply of cigarettes was raised to 21 [43].

The Global Alcohol Strategy is not legally binding, but its policy recommendations are cost-effective and are included in the WHO’s Best Buys for NCDs which includes alcohol and tobacco control measures [68]. These evidence-based and cost-effective measures are encapsulated in the WHO’s SAFER initiative, through which the country cases were assessed in this study but have yet to be adopted and implemented globally [24].

However, governance and leadership are hindered by policymakers’ lack of recognition of emerging but preventable public health issues. For example, while recent studies have pointed out the problem of binge drinking in Singapore, there have been no attempts to assess the potential of legally binding policies that can help prevent, if not minimize, the harmful effects of binge drinking, not only to the consumer but also to others around them, beyond increasing penalties for violating drunk driving regulations [69, 70]. Both countries are still hindered by a disease-based model for policymaking for NCDs instead of a risk-based public health model that is focused on disease prevention and health promotion [71].

The SDGs, the FCTC and the Global Alcohol Strategy provide a distinct policy window for further integration of disease prevention and health promotion not only in tobacco control but also in alcohol control. The relevant literature from low- and middle-income economies points out how interventions focused more on the detection and treatment of alcohol dependence rather than on harmful alcohol use, which is responsible for more alcohol-related harm, lead to delayed identification and care for harmful and hazardous drinkers [72]. Treatment gaps for alcohol use disorders even in a high-income economy like Singapore are due to delayed identification of the cases, often exacerbated by stigma [73].

An opportunity that both countries should consider is on implementing legally binding restrictions and regulations on AAPS, CSR and PPPs. There is pending legislation in the Philippines promoting and financially incentivizing CSR with no restrictions to health harmful industries, which has drawn opposition from public health advocates [74]. However, there is no robust evidence that the alcohol industry’s CSR initiatives aimed at reducing harmful drinking contribute to such goals and further complicated by a conflict of interest [74–76]. Such policy blind spots increase the alcohol industry’s power and influence, which are still evident even in institutions of global health governance [77].

### Limitations

This study has limitations that should be considered in the interpretation of its findings. It does not attempt to comparatively assess policy outcomes, stringency or effectiveness. Moreover, this study cannot provide a basis to generalize tobacco and alcohol control policies across middle- and high-income countries because of the small number of countries that were included for comparison.

### Conclusion

This study shows that using a health system-based scorecard for policy surveillance in alcohol and tobacco control can help set policy benchmarks, show the gaps and opportunities, and contribute to strengthening current policies. By using a public health law framework to assess tobacco and alcohol policies, we also identified neglected and new avenues for interventions. These opportunities include additional restrictions and regulations on alcohol marketing, financing of prevention (not just treatment) of tobacco and alcohol harms, and measures to protect policies from industry interference.

This in-depth comparative case study of two countries can be a useful framework to assess tobacco and alcohol control in other countries at various stages of economic and health system development. This study also provides opportunities for policymakers to assess a country’s progress over time vis-à-vis its national health agenda and global voluntary targets.
