# Corrigendum: Comparing Tobacco and Alcohol Policies From a Health Systems Perspective: The Cases of the Philippines and Singapore

**DOI:** 10.3389/ijph.2023.1606545

**Published:** 2023-10-30

**Authors:** Gianna Gayle Herrera Amul, Jean-Francois Etter

**Affiliations:** ^1^ Institute of Global Health, Faculty of Medicine, Université de Genève, Geneva, Switzerland; ^2^ Research for Impact Singapore, Singapore, Singapore; ^3^ School of Government, Ateneo de Manila University, Quezon City, Philippines

**Keywords:** tobacco control, health systems, alcohol control, Philippines, Singapore, health policy, public health law, policy surveillance

In the first published version of the article, there was an error. The word “improve” was mistakenly inserted between “marketing” and “corporate social responsibility programs” in the **Abstract** *Results* section. The word “improve” should be replaced by “and.”

A correction has been made to the **Abstract** *Results*:

“Results: Despite health system differences, both the Philippines (73.5) and Singapore (86.5) scored high for tobacco control, but both countries received weak and moderate scores for alcohol control: the Philippines (34) and Singapore (52.5). Both countries have policy avenues to reinforce restrictions on marketing **and** corporate social responsibility programs, protect policies from the influence of the industry, and reinforce tobacco cessation and preventive measures against alcohol harms.”

The authors apologize for this error and state that it does not change the scientific conclusions of the article in any way. The first published incorrect version of the article has been updated.

