# Supplementary material for: Comparing Tobacco and Alcohol Policies From a Health Systems Perspective: The Cases of the Philippines and Singapore
Source: Int J Public Health. 2022 Oct 13;67:1605050. doi: 10.3389/ijph.2022.1605050 (PMC9606809; doi:10.3389/ijph.2022.1605050)
Supplement: Supplementary file 1 [file DataSheet1.docx]

**Figure 1S. Literature Review on Alcohol and Tobacco Control in the Philippines and Singapore, 2020**

|  |
| --- |

**Table 1S. Tobacco excise taxation system in the Philippines and Singapore (Philippines and Singapore, 2020)**

|  | Excise tax (Philippines) | in USD (1 USD=PHP50.57) | Excise Tax (Singapore) | in USD (USD1=SGD1.39) |
| --- | --- | --- | --- | --- |
| Cigarettes | (per pack of 20)   - PhP 45 (2020) - PhP 50 (2021) - PhP 55 (2022) - PhP 60 (2023) - (5% indexation from 2024) | (per pack of 20)   - USD 0.89 - USD 0.99 - USD 1.09 - USD 1.18 | - SGD 0.427/gram or part thereof of each stick (2018) or 60% per pack of 20 | - USD 0.31 cent/gram or part thereof of each stick |
| Heated tobacco products | (per pack of 20)   - PhP 25 (2020) - PhP 27.50 (2021) - PhP 30 (2022) - PhP 32.50 (2023) - (5% indexation from 2024) | (per pack of 20)   - USD 0.49 - USD 0.54 - USD 0.59 - USD 0.64 | Banned product |  |
| Vapor products:  Nicotine salt or salt nicotine | (per mL)   - PhP 37 (2020) - PhP 42 (2021) - PhP 47(2022) - PhP 52 (2023) - (5% indexation from 2024) | (per mL)   - USD 0.73 - USD 0.83 - USD 0.93 - USD 1.03 | Banned product |  |
| Vapor products: conventional freebase or classic nicotine | (per 10 mL)   - PhP 45 (2020) - PhP 50 (2021) - PhP 55 (2022) - PhP 60 (2023) - (5% indexation from 2024) | (per 10 mL)   - USD 0.89 - USD 0.99 - USD 1.09 - USD 1.18 | Banned product |  |

**Table 2S. Alcohol taxation system in the Philippines and Singapore (Philippines and Singapore, 2020)**

|  | *Philippines* | | | *Singapore* | | |
| --- | --- | --- | --- | --- | --- | --- |
|  | *ad valorem* | Excise | in USD | Customs | Excise | in USD |
| Beer and other fermented alcoholic beverages (per liter) | - | - PHP 35 (2020) - PhP 37 (2021) - PhP 39 (2022) - PhP 41 (2023) - (6% indexation rate from 2024) | - 0.69 - 0.73 - 0.77 - 0.81 | (per litre of alcohol)  SGD 16 | (per litre of alcohol)  SGD 60 | 11.50 (Customs) + 43.07 |
| Wine | - | PHP 50 per liter | - 0.99 |  | (per litre of alcohol)  SGD 88 | 63.17 |
| Distilled spirits | 22% | (per proof liter)   - PHP 42 (2020) - PHP 47 (2021) - PHP 52 (2022) - PHP 59 (2023) - (6% indexation rate from 2024) | - 0.83 - 0.93 - 1.03 - 1.17 |  | (per litre of alcohol)  SGD 88 | 63.17 |

Note: Current exchange rates on xe.com were applied (USD1=PHP50.57 on 18 February 2020; USD1=SGD1.39 on 19 February 2020)

**Note 1S. Alcohol and Tobacco Licencing Schemes in Singapore and the Philippines**

Singapore has two main Customs schemes: (1) excise factory scheme which allows for the manufacturing and storing of dutiable goods, including tobacco and alcohol products in excise factories (2) licenced warehouse scheme which allows approved companies to store imported dutiable goods, including liquor and tobacco, for an indefinite period in a designated area (1) (2) (See Supplementary Table 5). In both schemes, the duty and goods and services tax are suspended. The excise factory scheme allows for the bottling, blending, compounding, varying intoxicating liquors in the licensed premises (1).

The Singapore Customs also requires prior approval for the import of apparatus or power-operated machinery for the manufacture of dutiable goods. (1). The annual licence fees for each dutiable product for the excise factory scheme and for the licensed warehouse scheme varies and ranges from SGD1,800 to SGD120,000 (see below). Under the licensed warehouse scheme, duty and goods and services tax are payable only when the goods are removed from the licensed warehouse for local use or consumption. In addition to the above, Singapore revised its Home Brewing Scheme in 2008, removing the licensing requirement for home-brewing of fermented liquors including beer and wine, with the rationale that removing the licensing requirement will “cut red-tape and align with best practices around the world (3).”

As regulated import products, the Philippines’ Bureau of Customs requires import permits for alcoholic beverages from the Food and Drug Administration (FDA) as well as the Bureau of Internal Revenue (BIR) and for tobacco products (cigars, cigarettes, e-cigarettes, and e-liquids) from the National Tobacco Administration (NTA) and the Bureau of Internal Revenue (4). The Bureau of Customs requires physical laboratory analysis/comment/evaluation on samples by the Bureau of Internal Revenue’s Laboratory Unit of every shipment of alcohol and alcohol products to determine its taxability and proper tax classification in the absence of acceptable certificate of analysis and/or material safety data sheet, product specification and/or product data/information(5). The Bureau of Customs also mandates that requests for the issuance of Bureau of Internal Revenue strip stamps or labels for alcohol or tobacco products can only be processed after the submission of proof of excise tax payment on the imported excisable articles by the application for the Authority to Release Imported Goods (ATRIG) (5). The Bureau of Customs also requires permits to import for tobacco and alcohol products, as well as for raw materials, apparatus or machines for the assembly and production of these products(5). Through the Philippine National Single Window System (NSW) led by the Bureau of Customs, applications for an electronic authority to release imported goods (eATRIG) can be done online (6).

Additionally, local governments in the Philippines are allowed to tax manufacturers, assemblers, repackers, processors, brewers, distillers, rectifiers and compounders of liquor, distilled spirits, and wines ranging from annual taxes of PHP 165 (less than PHP10,000 gross sales/year) to more than PHP24,375 but not more than 37.5% (for PHP6million in gross sales/year). Local governments are also allowed to tax manufacturers, producers, exporters, with factories, project offices, plants and plantations depending on the location of the principal office, factory, project office, plant, or plantation. Local governments are also mandated to regulate the sale, giving away or dispensing of any intoxicating product at any retail outlet. (7)

While Singapore has not yet ratified the Protocol, it has introduced a voluntary certification programme for all supply chain stakeholders, including importers, exporters, manufacturers, freight forwarders, warehouse operators, transporters and terminal operators called the Secure Trade Partnership (STP). The programme is modelled after the World Customs Organization (WCO) SAFE Framework of Standards for securing and facilitating global trade and “encourages companies to adopt robust security measures using a risk-based approach in their trading operations to improve global supply chain security(8).” In addition, Secure Trade Partnership Plus (STP-Plus), while still voluntary, allows companies with higher security measures to enter Mutual Recognition Arrangements (MRAs) with other Customs administrations which currently includes Canada, South Korea, Japan, China, Taipei, Hong Kong, US. Australia, New Zealand and Thailand (8). Several alcohol industry-related companies are under the STP programme, including Maybev (under Carlsberg Singapore) and the SUTL Corporation, while larger tobacco and alcohol companies are under the STP-Plus programme, including Asia Pacific Breweries (under Heineken) and British American Tobacco (9, 10). The certification programme under the Singapore Customs grants companies recognition as lower risk companies, with reduced inspections and expedited clearance under the MRAs(8).

**Table 3S. Licencing Schemes and Fees in Singapore**

|  | **Annual Licence Fee** |
| --- | --- |
| ***Excise Factory Scheme*** |  |
| Manufacture of cigarettes | SGD120,000 |
| Manufacture of tobacco other than cigarettes | SGD1,800 |
| Fermentation or manufacturing of ale, beer, stout, or porter with a projected annual production volume of 1.8 million litres or more | SGD43,200 |
| Fermentation or manufacturing of ale, beer, stout, or porter with a projected annual production volume of less than 1.8 million litres or more | SGD8,400 |
| Distillation, fermentation, or manufacturing of any other intoxicating liquor | SGD28,000 |
| Bottling, blending, compounding, or varying intoxicating liquors (in accordance with Section 66 of the Customs Act) | SGD7,600 |
| ***Licensed Warehouse Scheme*** |  |
| Projected potential duty of SGD1 million or less | SGD2,500 |
| Projected potential duty of more than SGD1 million but less than SGD10 million | SGS4,000 |
| Projected potential duty of SGD10 million or more | SGD21,000 |

**Table 4S. Evolution of earmarking of tobacco and alcohol taxes in the Philippines**

| **Legislation** | Revenue Allocation |
| --- | --- |
| **Tax Reform Code of 1997 (RA 8424)** | - 15% of incremental revenue from excise taxes on locally manufactured Virginia-type cigarettes are allocated to beneficiary provinces producing Virginia Tobacco (RA 7171) - 15% of incremental revenue from excise taxes allocated to provinces producing burley and native tobacco (RA 8240) |
| **Sin Tax Law of 2004 (RA 9334)** | - 15% of incremental revenue from excise taxes on locally manufactured Virginia-type cigarettes are allocated to beneficiary provinces producing Virginia Tobacco (RA 7171) - 15% of incremental revenue from excise taxes allocated to provinces producing burley and native tobacco (RA 8240) - 2.5% of incremental revenue from the excise tobacco and alcohol taxes for the Philippine Health Insurance Corporation (2005 to 2010) - 2.5% of incremental revenue from the excise tobacco and alcohol tax for the Department of Health as a trust fund for its disease prevention program (2005 to 2010) |
| **Sin Tax Reform 2012 (RA 10351)** | - 15% of incremental revenue from excise taxes on locally manufactured Virginia-type cigarettes are allocated to beneficiary provinces producing Virginia Tobacco (RA 7171) - 15% of incremental revenue from excise taxes allocated to provinces producing burley and native tobacco (RA 8240) - 80% of incremental revenue from excise tobacco and alcohol taxes earmarked for the National Health Insurance Program, the MDGs and health awareness programs, 20% earmarked for medical assistance and health enhancement facilities program |
| **Tobacco Tax Law 2019** | 50% of total revenue from excise tax on alcohol products   - 80% to PhilHealth for the implementation of the Universal Health Care Act (RA 11223) - 20% for medical assistance and the health facilities enhancement program   Total revenue from excise tax on tobacco products allocated   - 5% for provinces producing burley and native tobacco - 50% of total excise collection from tobacco products   - 80% to PhilHealth   - 20% for medical assistance and health facilities enhancement program   Total revenue from excise tax on heated tobacco products and vapor products   - 80% to PhilHealth - 20% for medical assistance and the health facilities enhancement program   15% of excise taxes on locally manufactured Virginia-type cigarettes for beneficiary provinces producing Virginia Tobacco |
| **Republic Act 11467** | 100% of total revenues collected from excise tax on alcohol products & on heated tobacco products and vapor products   - 60% for the Universal Health Care Act - 20% for medical assistance & the health facilities enhancement program - 20% for the Sustainable Development Goals |

Sources:

1. Singapore Customs. Excise Factory Scheme 2019 [Available from: <https://www.customs.gov.sg/businesses/customs-schemes-licences-framework/excise-factory-scheme>.

2. Singapore Customs. Licensed Warehouse Scheme 2019 [Available from: <https://www.customs.gov.sg/businesses/customs-schemes-licences-framework/licensed-warehouse-scheme>.

3. Singapore Customs. Relaxing rules for brewing liquor at home Singapore: Singapore Customs; 2008 [3:[Available from: <https://www.customs.gov.sg/-/media/cus/files/insync/issue03/tips/tips.html>.

4. Bureau of Customs. Regulated Import Products. In: Department of Finance, editor. 2019.

5. Bureau of Customs. Customs Memorandum Circular No. 34-2019 - Processing of BIR's authority to release imported goods (ATRIG). In: Department of Finance, editor. 2019.

6. Bureau of Internal Revenue. Revenue Memorandum Order No. 14-2014. In: Department of Finance, editor. 2014.

7. Congress of the Philippines. Republic Act No. 7160. Congress of the Philippines; 1991.

8. Singapore Customs. Secure Trade Partnership (STP) & STP-Plus 2020 [Available from: <https://www.customs.gov.sg/businesses/customs-schemes-licences-framework/secure-trade-partnership-stp>.

9. Singapore Customs. Secure Trade Partnership: STP Companies 2020 [Available from: <https://www.customs.gov.sg/-/media/stp-companies-090120---latest.pdf>.

10. Singapore Customs. Secure Trade Partnership: STP-Plus Companies 2020 [Available from: <https://www.customs.gov.sg/-/media/stpplus-companies-as-of-061119.pdf>.
